# Perceived usability and acceptability of the My-Hip Fracture risk communication tool from the perspective of academic clinicians

**DOI:** 10.1016/j.pecinn.2024.100360

**Published:** 2024-11-26

**Authors:** Erin L. Hommel, James P. Flaherty, Caitlin R. Aguirre, Amber S. McIlwain, Monique R. Pappadis, Pete Wegier, Peter Cram

**Affiliations:** a School of Medicine, The University of Texas Medical Branch, 301 University Blvd, Galveston, TX 77555, USA; b School of Public and Population Health, The University of Texas Medical Branch, 301 University Blvd, Galveston, TX 77555, USA; c Sealy Center on Aging, The University of Texas Medical Branch, 301 University Blvd, Galveston, TX 77555, USA; d Institute of Health Policy, Management and Evaluation, The University of Toronto, 155 College St 4^th^ Floor, Toronto, ON M5T 3M6, Canada; e Department of Family and Community Medicine, The University of Toronto, 500 University Avenue, Toronto, ON M5G 1V7, Canada; f Humber River Health Research Institute, 200 Church Street, Toronto, ON M9N 1N8, Canada; g School of Medicine, University of Maryland, 655 West Baltimore St, Baltimore, MD 21201, USA

**Keywords:** Hip fracture, Prognosis, Risk communication, Electronic health

## Abstract

**Objective:**

We evaluated the usability and acceptability of My-Hip Fracture (My-HF), a web application that assists providers in delivering individualized prognostic information to patients hospitalized for hip fracture (HF).

**Methods:**

We observed a sample of 16 clinicians as they navigated My-HF. We then administered a structured questionnaire and conducted semi-structured interviews to explore participants' opinions about the app's content and the feasibility of incorporating the app into clinical workflows.

**Results:**

Clinicians required a median of 2-min and 45 s to navigate through the app. Nearly all participants indicated that My-HF was easy to use and would be useful for their practice. About half of participants had suggestions for additional useful peri-operative content. A few expressed concerns about communicating mortality risk. About half expressed concerns about how My-HF might be integrated into existing clinical workflows.

**Conclusions:**

Though clinicians rated My-HF high on usefulness in a structured usability questionnaire, qualitative interviews identified a number of suggestions for optimizing integration into clinical practice. Creating shared goals, establishing a decision coach, and developing a framework of communication across care settings could facilitate integration of My-HF by the multidisciplinary HF team.

**Innovation:**

My-Hip Fracture is a unique web application which provides personalized prognostic information to patients and families after HF. My-HF has potential to facilitate informed decision-making between clinicians and patients, but adaptations will be necessary to enhance its usability.

## Highlights

• My-Hip Fracture app helps clinicians deliver prognostic information after hip fracture.
• We had clinicians evaluate the usability and acceptability of My-Hip Fracture.
• Despite high usability ratings, clinicians questioned the feasibility of integration.
• Creating shared communication goals and using a decision coach could help.
• Mechanisms to facilitate communication across time and settings are also needed.

## 1 Introduction

Approximately 250,000 Americans and 14 million persons worldwide experience a low trauma hip fracture (HF) annually [1,2]. Morbidity and mortality after HF are high, with 30-day mortality of 5 % to 10 % and 1-year mortality of 20 % to 30 % [3,4]. Fewer than 50 % of persons who survive HF regain pre-fracture function [5].

Patients and families are often unaware of the seriousness of HF. Most patients believe they will recover within a few weeks and are surprised to learn that functional recovery may be protracted and incomplete [[6], [7], [8], [9]]. Unrealistic recovery expectations can lead to disappointment, dissatisfaction, and regret. Conversely, improved prognostic communication could increase satisfaction and facilitate conversations around advanced care planning and patients' ability to live independently [10].

Most educational tools for HF patients and families focus on rehabilitation and secondary fracture prevention [[11], [12], [13]] with prognostic information limited to generic statements about slow recovery. Numerical risks, if stated, are usually population estimates for mortality or recovery, not personalized risk estimates. Risk calculators for clinicians to estimate short-term prognosis after HF exist [14,15] but are not designed for direct communication with patients and families. This limits their utility in shared decision-making for recovery and end-of-life care.

Shared decision-making models, such as the Interprofessional Shared Decision Making Model (IP-SDM), outline key stakeholders, system-level influences, and action steps for effective, informed decision making [16]. The decision-making process requires careful information exchange, evaluation of values and preferences, and determination of choice. The Revised Program Theory, devised to support the IP-SDM framework, further defines key factors in the effectiveness of shared decision-making [17]. These factors include stakeholders' willingness to engage in the process, patient (and family) anxiety and trust in the provider, time required to participate, and availability of external support, including decision aides.

We developed an informational tool to facilitate shared decision-making after HF. My-Hip Fracture (My-HF) is a web-based application for physicians or advanced practice providers to use at the bedside with patients and families to explain the hip injury and possible short-term outcomes. Following an integrated knowledge translation approach [18], we tested paper prototypes with patients and families [19], then developed and iteratively refined the web application, which uses clinician-entered patient information to calculate personalized risk estimates and generate a patient-friendly educational report. Following the IP-SDM model, My-HF is specifically designed to facilitate exchange of knowledge and expression of values and preferences as critical steps in shared decision-making after HF.

We previously described the usability of My-HF from the perspective of patients experiencing acute HF and their families [19], but the usability and acceptability of My-HF among clinicians is unknown. This study examines the usability of My-HF among a sample of academic clinicians and assesses their feedback on its feasibility for integration into routine clinical workflows. Feasibility, which may be limited by perception of time and the patient's or family's capacity to participate in the exchange of information, is recognized as a critical hurdle within the Revised Program Theory and IP-SDM model.

## 2 Materials and methods

We conducted a mixed-method study using a structured usability questionnaire and semi-structured interviews to examine the usability and acceptability of My-HF among clinicians familiar with HF care. The study was approved by the Institutional Review Board at the University of Texas Medical Branch at Galveston (UTMB) on May 11, 2022 (IRB #22–0027).

### 2.1 Overview of My-HF

We developed My-HF for clinicians to use with a patient or family member during an acute hospitalization for HF. My-HF is designed to facilitate post-operative conversations regarding the HF injury and expected 30-day prognosis; it is not a pre-operative decision aide. We focused on managing patient and family expectations for recovery after HF rather than decision making about surgery for two reasons. First, surgical repair is standard-of-care in all but a select few extremely high-risk patients. Second, we wanted to facilitate patient centered, informed decisions about advanced care planning and post-acute care after hip-fracture while managing patient and family surprise and regret along the recovery journey.

My-HF was developed following best practices in human-centered design [20]. The app requires clinicians to input eight demographic and seven clinical variables (**Supplemental File: Appendix 1**) to generate a personalized patient report that includes information about HF anatomy, surgical repair performed (if any), predicted risk of 30-day severe complications, predicted risk of 30-day mortality, and anticipated post-discharge care needs. Prognostic estimates are calculated using a statistical model adapted from the American College of Surgeons National Surgical Quality Improvement Program (ACS-NSQIP) participant use data files [21].

The report is available in My-HF and in a portable document format (PDF) that can be provided to the patient or family (see **Supplemental File: Appendix 2** for an example). Risk estimates for serious complications and mortality include a pictorial representation of the outcome, graphical depiction of the patient's risk compared to an average HF patient, and written explanation of the patient's risk. Reports were created with attention to patient health numeracy and literacy [[22], [23], [24], [25]].

While hip fracture care requires the skills of a large interdisciplinary team—including physicians, nurses, social workers/case managers, and physical and occupational therapists—this analysis focuses on the information exchange facilitated by My-HF between patients and their physician or advanced practice provider. Given the complexity of the clinical inputs and outputs from My-HF, as well as our experiences as practicing clinicians who frequently care for patients with HF, we believed that physicians and advanced practice providers would have the greatest credibility and skill to deliver the necessary information, thereby reducing potential implementation barriers identified in the Revised Program Theory and IP-SDM model.

### 2.2 Study setting

We conducted this study at an academic health system in Southeast Texas. Its primary hospital is a level 1 trauma center and quaternary referral center for nine surrounding counties. Annual HF volume exceeds 250 patients. A multidisciplinary team, including anesthesiology, orthopedic surgery, primary care, nursing, physical and occupational therapy, and social work, provides comprehensive HF care. The orthopedic and primary care co-management team follows institutional HF protocols for pre-operative medical optimization and post-operative recovery consistent with best practice standards [26].

### 2.3 Participant recruitment

We recruited a convenience sample of faculty and residents/fellows in orthopedics, family medicine, internal medicine, and geriatrics using emails and announcements at departmental meetings. Clinicians were eligible to participate if they had provided recent care for HF patients and were not on our research team. We aimed for a representative sub-sample of three to five participants from each department (12 to 20 participants total) to permit qualitative data saturation [27].

### 2.4 Study protocol

After providing informed consent, each participant completed a demographic questionnaire that included age, sex, academic appointment (resident, fellow, faculty), degree (Medical Doctor [MD], Nurse Practitioner [NP]), specialty, years in practice (time since terminal degree), and estimated number of HF patients cared for in the prior year.

We then introduced participants to the rationale and design for My-HF and observed participants navigating it on a tablet using a simulated patient scenario (**Supplemental File: Appendix 3**). We recorded the time participants needed to navigate data entry and captured technical concerns using field notes. Data were collected and managed using REDCap.

After participants navigated My-HF, we conducted an audio-recorded semi-structured in-person interview to capture feedback on the usability of the app. Participants also completed an 11-item usability questionnaire adapted from the 21-item Mobile Health (mHealth) App Usability Questionnaire (MAUQ) for interactive mHealth apps used by healthcare providers [28]. Responses were recorded using the MAUQ's suggested 7-point Likert scale (1 = strongly disagree, 7 = strongly agree). Greater scores indicate greater perceived usability.

### 2.5 Quantitative analysis

We reported participant demographics as medians with the interquartile range or as raw counts with percentages, data entry time as the median time in minutes and seconds with the interquartile range, and technical application failures as raw counts. For each item of the usability questionnaire, we reported the median and interquartile range based on the 7-point Likert scale. Due to the small sample size, the infrequent use of many Likert scale responses, and the skew of results, we also collapsed questionnaire responses by question into three categories: disagree (responses 1–3), neither agree nor disagree (response 4), and agree (responses 5–7). We depicted the number of responses per category to better express the range of participant responses.

### 2.6 Qualitative analysis

Interviews were transcribed using Otter.ai™. ELH reviewed transcripts to ensure deidentification and correct transcription errors. We then performed thematic analysis using a combined deductive-inductive approach [29]. The deductive themes of preferred content, perceived feasibility of use, and overall impression were informed by the IP-SDM elements of information exchange, exchange of values and preferences, and feasibility. These themes also allowed exploration of the Revised Program Theory factors of perception of patient and family capacity, anxiety, and trust, as well as perception of time and perception of capacity to access external support. Sub-themes within the main themes of preferred content and perceived feasibility of use emerged inductively. Table 1 depicts the interrelationship of the IP-SDM framework, the Revised Program Theory mechanisms, the deductively derived themes, and the inductively derived sub-themes. Three coders (ELH, JPF, CRA) independently coded the first three transcripts and collaboratively developed the codebook for the thematic analysis. Remaining transcripts were independently coded by two coders (JPF, CRA). At team meetings, we used an interactive process to share coding and challenge interpretations, with a third coder (ELH) available to resolve coding discrepancies. To aid understanding, we used non-specific terms such as “more than half,” “about half,” “less than half,” and “few” to convey the regularity of the responses. To enhance application, we included participants' field of practice, though other descriptors were removed to preserve anonymity. Analyses were performed using NVivo Release 1.0™.

**Table 1:** Mapping of Themes to the IP-SDM Framework.

IP-SDM Elements	Revised Program Theory Mechanisms	Deductive Themes	Inductive Sub-themes
Information Exchange Values/Preferences	• Perception of patient and family capacity • Anxiety • Trust	Preferred Content	• Peri-operative/Post-operative Instruction • Rehabilitation/Recovery • Risk of complication/death
		Overall Impression	
Feasibility	• Perception of Time • Perception of capacity to access external support	Perceived Feasibility of Use	• Time-required • Who should administer My-HF? • Timing of when to use My-HF during the HF episode • Electronic health barriers • Universal adoption of My-HF
		Overall Impression	

## 3 Results

### 3.1 Participant demographics

We recruited 16 clinicians (15 MDs, 1 NP) with a median age of 39.5 years (interquartile range [IQR] 29–44) and median years in practice of 5.5 (IQR 3–14) (Table 2). Participants included seven trainees and nine faculty. Participants were split evenly between family medicine, internal medicine, geriatrics, and orthopedics specialties.

**Table 2:** Participant demographics.

	Participants (*N* = 16)
**Age in years, median (IQR**⁎**)**	39.5 (29–44)
**Years in practice, median (IQR**⁎**)**	5.5 (3–14)
**Sex, n (%)**	
Male	6 (37.5)
Female	10 (62.5)
**Academic Appointment, n (%)**	
Residents/Fellows	7 (43.8)
Faculty Members	9 (56.3)
**Degree, n (%)**	
Doctor of Medicine	15 (93.8)
Nurse Practitioner	1 (6.3)
**Specialty, n (%)**	
Family Medicine	4 (25.0)
Internal Medicine	4 (25.0)
Geriatrics	4 (25.0)
Orthopedics	4 (25.0)
**Number of Patients seen with Hip Fracture (last 12 months)**	
<20	10 (62.5)
20–40	3 (18.8)
>40	3 (18.8)

⁎ IQR = Interquartile range.

### 3.2 Technical aspects of using My-HF

Median time needed to enter required data elements was 2 min and 45 s (IQR 2 min 13 s to 3 min 49 s). One participant had to repeat their session when unexpected app maintenance caused application failure.

### 3.3 Usability questionnaire results

**Fig. 1:**
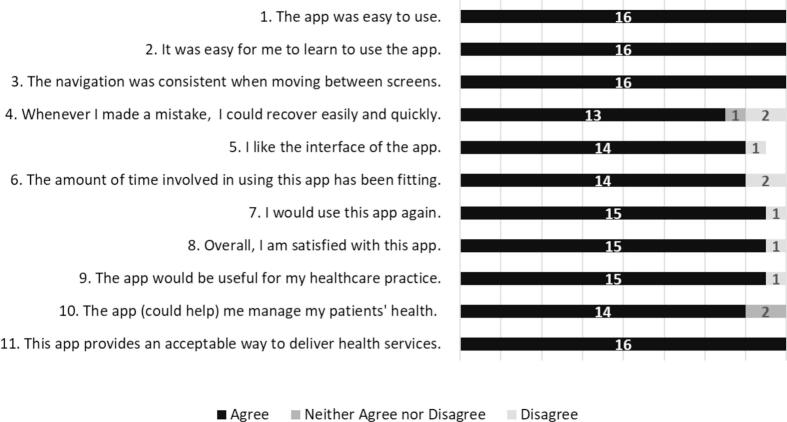
Usability Questionnaire Responses. Responses are collapsed from the original 7-point Likert scale into the three categories of agree, neither agree nor disagree, and disagree. Response are available from all 16 participants for each question, except item 5, for which a single response was missing.

Participants found My-HF useful across all domains of the usability questionnaire (Fig. 1). All participants (100 %) found My-HF easy to use (question 1: median 6, IQR 6–7) and learn (question 2: median 7, IQR 6–7). All participants also agreed that navigation was consistent across the app (question 3: median 7, IQR 6–7) and that it was an acceptable method to deliver health services (question 11: median 7, IQR 6–7). Two participants (12.5 %) reported difficulty recovering after making errors (question 4: median 7, IQR 6–7) and were dissatisfied with the time required for use (question 6: median 6, IQR 6–7). Almost all participants (93.8 %) liked the interface (question 5: median 7, IQR 5–7), were satisfied with the overall experience (question 8: median 6, IQR 6–7), agreed that My-HF would be useful for their practice (question 9: median 7, IQR 6–7), and indicated they would use it again (question 7: median 7, IQR 6–7). Fourteen participants (87.5 %) reported that My-HF could be used to help manage a patient's health (question 10: median 7, IQR 6–7).

### 3.4 Qualitative analysis results

Qualitative analysis of the semi-structured interviews was organized around the deductive themes of preferred content, perceived feasibility of integration, and overall impression of the app.

#### 3.4.1 Preferred content

The theme of preferred content allowed us to examine the participant's perception of the patient's and family's capacity to receive information as presented in My-HF. Perceived capacity to receive information is a critical mechanism identified in the Revised Program Theory that may facilitate or impede shared decision-making. This theme also permitted exploration of how anxiety or trust within and between the clinician and patient/family might influence preferences for information exchange after an acute HF. We identified three sub-themes related to content: peri-operative/post-operative instruction, rehabilitation/recovery, and risk of complication/death.

##### 3.4.1.1 Peri-operative and post-operative instruction

Participants appreciated the information about fracture type and surgical repair. An internal medicine representative described the benefit of providing this information in the app:

I like the explanation of anatomy and the kind of details regarding the procedure because oftentimes patients will ask their primary team, which includes medical students and residents and attendings. And if we're a general medicine floor, we don't necessarily know the exact steps of the surgical procedure.

About half of participants desired additional content addressing how patients can reduce the risk of serious complications. A representative of orthopedics explained:

I think a lot of [patients] are going to be unsure about what to do with the information … and they're immediately [going to ask] ‘how do I prevent this?’

Participants from orthopedics and internal medicine were more likely to suggest that My-HF include additional patient education on immediate post-operative care, such as osteoporosis treatment and falls prevention, pain control strategies, wound care, and the importance of taking blood thinners.

##### 3.4.1.2 Rehabilitation and recovery

Participants appreciated the ability to personalize the expected discharge destination and found value in the inclusion of expectations for this destination. A representative of orthopedics described the need for this information:

I like that [My-HF] covers some of the things [patients] might be doing if they're going to a rehab place. Because a lot of times we just say, “Ask your physical therapist. I'm not sure exactly what they're going to be doing.” So I think it's got good information.

A few participants suggested adding information about anticipated functional needs beyond the post-acute care period, with predictions about recovering ambulation and ability to live independently. While representatives of geriatrics were more likely to request this content, a representative of internal medicine also highlighted common patient and family concerns during recovery that My-HF could help address:

I think the other thing would be like, what's next? Like, if you put [patients] into [a skilled nursing facility] or rehab, is that the end? Are they okay to go home? Or do they need to stay in the [skilled nursing facility] permanently? Or do they just need more rehab?

##### 3.4.1.3 Risk of complication and death

About half of participants described the information on risk of serious complications as important for patients and families to appreciate the severity of HF and set realistic expectations. Participants similarly described the information on mortality risk, recognizing its importance in setting care goals. A representative of internal medicine explained:

When a patient knows about what's going on, and what the procedure entails, they can make better decisions. It honestly helps with care goals, too.

A few participants, however, found the inclusion of mortality risk to be of questionable benefit or even dangerous. These participants wanted to eliminate the content altogether or change its mechanism of conveyance to soften the message. A representative of family medicine noted:

A lot of people are very uncomfortable with discussing death.

Additional representative quotes on the pros and cons of discussing risk of serious complications or death are presented in Table 3. In general, participants representing geriatrics were in favor of including straightforward language about prognosis, whereas participants representing internal medicine and family medicine were less comfortable discussing morbidity and mortality after HF. Representatives of orthopedics generally assumed a more neutral opinion on the matter. Only one internal medicine representative questioned the accuracy of the risk predictions.

**Table 3:** Representative Quotes on Content Regarding Risk of Complication and Death⁎

PROS	
Specialty	Quote
Family Medicine	“I think it would be a good way for us to make sure that they understand, you know, what the complications are postoperatively.”
Orthopedics	“I mean, I don't know. Like, if I knew how to have this conversation in a way that was appropriately aggressive versus not aggressive, I would have this conversation, but I don't. That being said, I think it takes the guesswork out of it…Understanding the risk of death is, you know, I think it's important…I think that every patient should be aware of, you know, their health status and understand what's going on with their body. So if we could give this to people, and they could tolerate this, then I think that it would be really valuable. And to be honest, people usually tend to handle bad news better than I expected they would.”
Orthopedics	“I mean, on one hand, it's aggressive (reflecting on the skull and crossbones). But also, I think that's one of the really important things that people need to know. Regardless of what you do. Yeah, that's something you're looking at six months or a year out as well…I think the really good stuff is high risk things and death is obviously high risk. But for surrogate decision makers and stuff, it helps them appreciate the severity of a low energy, hip fracture.”
**CONS**	
**Specialty**	**Quote**
Internal Medicine	“I mean, in my mind, to be honest with you, I want to focus on what the family can do, what they can control, and how they can help. I think it's good to have a little information about risk. But the reality is, is that you know, the way to prevent blood clots, the way to prevent these things are to get up and move. There's not a lot on here about that… So in my mind, I would prefer to focus on what to do, and how they can prevent these things, rather than about their risk, which is good, but also not, you know, not super helpful on how they can prevent those things.”
Family Medicine	“So for your individual risk of death, I might… a lot of people are very uncomfortable with discussing death. And so I might suggest for this one, I might not have a picture like that skull and crossbones. You might find family members to be angry if you don't soften it a little. Yeah. And then you know, then your managing, not the patient's problem but the patient being sad afterwards.”
Family Medicine	“Death is scarier. So the question I guess goes back to, like is it right to communicate that seriousness?”

⁎ Each quotation included represents the perspective of a unique participant.

#### 3.4.2 Perceived feasibility of integration

The theme of perceived feasibility of integration allowed us to examine the participant's perception of time as a facilitator or barrier to information exchange for shared decision-making after HF. We also analyzed the participant's perceived need for and capacity to access external support. These elements of the Revised Program Theory reflect the interprofessional focus of the IP-SDM model and the necessary step to address feasibility of information exchange. In total, we identified five sub-themes related to feasibility of integrating My-HF into routine clinical workflows.

##### 3.4.2.1 Time required

More than half of participants mentioned time when considering the feasibility of integrating My-HF into workflows, but opinions varied on whether the time required was a barrier, facilitator, or neither. A few considered time a neutral factor, indicating that even though clinicians are resistant to new steps in their workflow, My-HF is less cumbersome than other tools used. Others, including a representative of family medicine, were more positive, describing use of My-HF as brief and straightforward:

It didn't really take more than like two or three minutes. It's all information we know. So I don't think it would be difficult to have that done. Of course, you may have people that are resistant. Because they don't want to do anything else. But it really doesn't require too much time.

A few participants identified time as a distinct barrier to use, finding the data entry tedious. A representative of geriatrics explained:

Right now, it was fairly quick. But still, what was it, five minutes? ten minutes? extra that it took that most people will not be willing to devote.

Overall, there were no trends by specialty in perception of time as a barrier, facilitator, or neutral factor.

##### 3.4.2.2 Who should administer my-HF?

Participants expressed uncertainty over who on the healthcare team was best positioned to administer My-HF, as illustrated by a representative of orthopedics:

I felt like I was spending a lot of time answering questions for knowledge that is already on the chart and accessible for somebody else. [Therefore] I think it is something that I would request my team do for me.

A few participants suggested assigning the task to nurses or social workers. A representative of family medicine explained:

I would make the nurses responsible for that. Not the doctor. Because if it's the nurses who are responsible, it will be done. If it's a doctor, it won't.

In general, representatives of family medicine and orthopedics were more likely to recommend a non-physician team member own the information exchange, while representatives of geriatrics or internal medicine were more likely to agree with physician delivery but questioned if a surgeon or primary care physician was more appropriate. Regardless of who completed My-HF, most participants believed a dedicated owner would increase the probability of completion.

##### 3.4.2.3 Timing of when to use My-HF during the HF episode

While My-HF was designed to facilitate post-operative conversations regarding the HF injury and expected 30-day prognosis, a few participants, including an internal medicine representative, believed My-HF could add value to the pre-operative conversation:

I almost feel like [the app] should be pre- and post-op … Like the anatomy risk factors and the surgery itself, it seems would be really helpful upon admission. And then the rest of it would be more helpful after the surgery, right prior to discharge.

About half of participants, including a representative of geriatrics, felt that revisiting My-HF during post-acute follow-up visits would promote further discussion:

I think this would be great to incorporate in the [skilled nursing facility] … to be able to send it with the [patient] because I think there's an environment at least to educate the caregivers. And then, having something that we could even access [in the outpatient clinic], too, if there are questions.

These perspectives highlight the continuum of multidisciplinary shared decision-making as seen in the IP-SDM model.

##### 3.4.2.4 Integration with the electronic medical record

My-HF exists as a web-based application external to the electronic medical record (EMR). More than half of participants discussed this as a potential barrier to integration. A representative of orthopedics suggested that integration into the EMR could facilitate use:

Obviously, I think, if it was integrated [into the EMR] where 80–90 % of it's just auto populated, then it would definitely be filled out. You probably would have a higher compliance rate.

A few participants expressed uncertainty about accessing My-HF at the bedside, questioning the preferred device or how to bookmark the app for ease of access, but a representative of geriatrics described the appeal of using My-HF at the bedside:

Having it at bedside would be really great. Especially because we have the computers in the room as well. It's easy for you to just open up a link and just go through to the website.

Integration into the EMR is an important aspect of external support that can either facilitate or hinder information exchange in shared decision-making.

##### 3.4.2.5 Universal adoption of My-HF

About half of participants believed it would be difficult to get My-HF into routine clinical workflows because it had not been part of the standard-of-care historically. A representative of internal medicine discussed the importance of buy-in for the adoption of new applications:

I think there's always buy-in with these kinds of things, like getting people who have done this their way for the past 20 years to buy into a new application. I think younger people are used to using applications so we're more open to adopting these kinds of resources. But if it's another year before you treat a hip fracture patient and no one has reminded you that this resource is there, it's going to be a problem.

These participants suggested various mechanisms to increase the feasibility of universal adoption, including clinician education and embedding My-HF into existing HF clinical guidelines and risk prediction tools.

#### 3.4.3 Overall impression

Participants were asked to share their overall experience with My-HF, allowing us to identify specific aspects of My-HF that contribute to its usability for information exchange after an acute HF. First, participants found the educational content well-structured and comprehensive. A representative of family medicine deemed these qualities particularly beneficial for patients and families, who may not realize the seriousness of HF:

It's going to be a great offering to our patients. It helps [patients and families] appreciate the severity of a low energy hip fracture.

Second, participants found My-HF's personalized information an advantage over generic education. An internal medicine representative explained that receiving customized information is more meaningful:

I think things mean more to people when they've been customized … like this is applicable to you as opposed to when I hand you a generic thing.

Third, participants considered the PDF summary an important product for patients and families to review in future encounters. A representative of internal medicine explained:

Having something that [patients and families] can take home and read makes a big difference for a lot of those folks.

Finally, participants believed My-HF would be educational for clinicians and not just patients and families. Another internal medicine representative valued learning more about the risks associated with HF:

I appreciate it. Like the way it presented [information] made me think about things. I think it was educational for me to a degree because I've not looked at the real risk of this.

## 4 Discussion and conclusion

### 4.1 Discussion

We examined clinicians' perceptions of the usability and acceptability of My-HF, a risk communication tool designed to facilitate information exchange for shared decision-making after HF and to help better manage patient and family emotions such as surprise and regret during recovery. Quantitative analysis of the usability questionnaire suggested that My-HF is easy to use and helpful to clinicians when counseling patients and families after HF. Qualitative analysis confirmed My-HF's usefulness in providing needed education about HF but also uncovered opportunities for improvement when planning broader implementation. Comparing quantitative and qualitative usability data before widespread adoption of mobile health resources increases the likelihood of successful implementation by using iterative and convergent mixed methods [30]. We discuss important lessons learned using this approach to study the usability of My-HF.

We applied the IP-SDM model to this research because of the complexity of shared decision-making after HF. The inpatient and post-acute HF care team included primary care physicians, surgeons, physical and occupational therapists, nurses, and social workers spread across hospital, rehabilitation, home, and community settings. To facilitate advanced care planning and goals for post-acute care, clinicians should understand and be able to communicate the seriousness of the HF injury and set realistic expectations for patients and their families regarding recovery. Even in this study, which focuses on information exchange between experienced physicians or nurse practitioners and patients during an inpatient post-operative encounter after HF, we identified a lack of common understanding about HF that is critical for guiding patients and families through the decision-making process [16].

While some clinician participants were uncertain about prognosis after HF and appeared to lack knowledge, others were well-informed about prognosis but uncomfortable discussing mortality with patients and families. A qualitative study of clinicians who care for HF patients found substantial physician discomfort in discussing mortality; physicians struggled to initiate the conversation despite understanding the seriousness of HF, mirroring our findings [31]. Interestingly, family medicine and internal medicine physicians in our study expressed more discomfort with having straightforward prognostic conversations compared to geriatricians and orthopedic surgeons. A recent scoping review examining physician factors influencing prognostic discussion also found that family medicine and internal medicine physicians tend to be more optimistic about patient recovery [32]. Many physicians are concerned that truthful prognostic discussions will jeopardize patient hope and the patient-physician relationship, but research suggests that most patients desire to understand prognosis and that knowing does not increase their distress [33].

Another study describing a model for multidisciplinary HF care identified four key components for success: defined roles and responsibilities, collaborative leadership, a process for information transfer, and shared goals [34]. In our study on the usability of My-HF for information exchange after HF, we found that internal medicine physicians and orthopedic surgeons were primarily focused on immediate post-operative care, while geriatricians tended to prioritize longer-term recovery and prognosis. This suggests that My-HF, in its current form, could be used for different purposes by different team members to achieve varying goals. If the aim of My-HF is to promote shared decision-making for rehabilitation and recovery, establishing shared goals around risk communication and emphasizing longer-term rehabilitation and recovery needs will be a critical first step. Creating this mutual understanding would help align agreement on the necessary content of the app.

The IP-SDM model highlights that care decisions are made within the context of the organization's multidisciplinary team (*meso* level) and within the broader health system (macro level) [16]. Although our study focused on information exchange within the inpatient setting, participants aptly identified the need to extend shared decision-making across the entire care continuum, from pre-operative to post-operative, inpatient to outpatient, and post-acute care facility to home. This requires clearly defining the multidisciplinary HF care team across different care environments. Amending the IP-SDM model to more explicitly address the complexity of shared decision-making across time and care settings could assist others in designing more successful approaches.

It is incredibly difficult to institute a cohesive shared decision-making approach across clinicians, time, and settings. Within the IP-SDM model, patients and families could facilitate this ongoing conversation, but we believe success hinges on the engagement of the decision coach [16]. In our study, we did not suggest or acknowledge a support role in the process of risk communication or shared decision-making after HF. However, orthopedic surgery and family medicine participants readily identified this as a key strategy to enhance uptake of My-HF, citing various concerns for physicians to feasibly integrate the app into existing workflows. Appointing a well-educated and appropriately skilled team member for information exchange and facilitation of shared-decision making across time and settings would likely increase buy-in.

Importantly, fracture liaison services exist as a model to improve implementation of post-fracture best practice standards [35]. Frequently lead by nurses or advanced practice providers, these services include patient and family education around diagnosis, treatment, and prognosis [36]. While fracture liaison services have not historically focused on planning rehabilitation, recovery, or end-of-life care, we believe the fracture liaison service coordinator is primely positioned to serve as the decision coach in shared decision-making following HF.

Our investigation has limitations. We were limited to a convenience sample of 16 clinicians at a single academic institution. Our participants had experience with HF care through standardized institutional protocols. Participants' experience with My-HF may not be generalizable to a community hospital setting or sites less experienced in care of HF patients. In this study, we focused on information exchange between physicians or advanced practice providers and patients. While we propose potential roles for other interdisciplinary team members in shared decision-making after HF, studies are needed to assess usability by these clinicians. Finally, we have not studied the impact of My-HF on relevant clinical and patient-reported outcomes linked to shared decision-making, such as advanced care planning and goal setting for post-acute care. This will be an important next step in the My-HF validation process.

### 4.2 Innovation

The My-HF web app is a useful, well-structured, and uniquely personalized risk prognostication tool designed to ease communication with patients and families following an acute HF. We employed a combined quantitative and qualitative analysis to comprehensively examine the app's usability in promoting information exchange and encouraging shared decision-making. Modifications to the app's content and integration process, based on feedback from the multidisciplinary care team, will enhance the tool's future applicability. Engaging a decision coach, such as a fracture liaison service coordinator, in the administration of My-HF could further facilitate ongoing shared decision-making for recovery and advanced care planning throughout the HF episode of care.

### 4.3 Conclusion

Communicating risk and engaging patients and families in shared decision-making after HF is challenging. Clinicians vary in their comfort, availability, and skill in delivering prognostic information to patients. In addition, advanced care planning after HF must be facilitated across multiple clinicians, across time and care settings.

My-HF offers a framework to initiate difficult conversations about personalized risk of morbidity, mortality, and prognosis after HF. But its success will depend upon fostering a mutual understanding and shared goals within the multidisciplinary team and expanding discussions beyond the hospital setting.

## Funding details

Dr. Erin Hommel and Dr. Monique Pappadis are supported by the Claude D. Pepper Older Americans Independence Center Award (#P30-AG024832), which is funded by the National Institutes of Health/National Institute on Aging (NIA).

## CRediT authorship contribution statement

**Erin L. Hommel:** Writing – review & editing, Writing – original draft, Project administration, Methodology, Investigation, Funding acquisition, Formal analysis, Data curation, Conceptualization. **James P. Flaherty:** Writing – review & editing, Writing – original draft, Formal analysis, Data curation. **Caitlin R. Aguirre:** Writing – review & editing, Writing – original draft, Formal analysis, Data curation. **Amber S. McIlwain:** Writing – review & editing, Writing – original draft, Visualization. **Monique R. Pappadis:** Writing – review & editing, Supervision, Methodology, Conceptualization. **Pete Wegier:** Writing – review & editing, Supervision, Methodology, Conceptualization. **Peter Cram:** Writing – review & editing, Writing – original draft, Supervision, Resources, Methodology, Conceptualization.

## Declaration of competing interest

The authors declare the following financial interests/personal relationships which may be considered as potential competing interests:

Erin Hommel reports financial support was provided by National Institute of Health. Monique Pappadis reports financial support was provided by National Institute of Health. If there are other authors, they declare that they have no known competing financial interests or personal relationships that could have appeared to influence the work reported in this paper.

## Appendix A Supplementary data

Supplementary material Image 1
