# Supplementary material for: Perceived usability and acceptability of the My-Hip Fracture risk communication tool from the perspective of academic clinicians
Source: PEC Innov. 2024 Nov 26;5:100360. doi: 10.1016/j.pecinn.2024.100360 (PMC11650269; doi:10.1016/j.pecinn.2024.100360)
Supplement: Supplementary file 1 — Supplementary material [file mmc1.docx]

**Appendix 1: Variables Required for My-HF Risk Calculation**

| **Variable** | **Details** |
| --- | --- |
| Name | For identification purposes only |
| Medical Record Number | For identification purposes only |
| Date of Birth | Entered as MM/DD/YYYY, to calculate age as continuous variable |
| Sex | Male or Female |
| Height | Entered In inches or meters |
| Weight | Entered In pounds or kilograms |
| Surgical Procedure (if any) | Options: No Surgery - comfort care, No Surgery - non-operative fracture, Internal Fixation - cancellous screws, Internal Fixation - dynamic hip screw, Internal Fixation - intramedullary nail, Partial Hip Replacement, Total Hip Replacement |
| Functional Status | Options: Independent, Partially Dependent, Totally Dependent |
| Ascites | Yes/No |
| Systemic Sepsis | Yes/No |
| Disseminated Cancer | Yes/No |
| Diabetes | Options: No Diabetes, Diabetes No Insulin, Diabetes with Insulin |
| Congestive Heart Failure | Yes/No |
| Severe COPD | Yes/No |
| Acute Renal Failure | Yes/No |

**Appendix 2: Example My-Hip Fracture Patient Web-Based Report**

1. **Hip Joint Anatomy and Fracture Risk Factors**


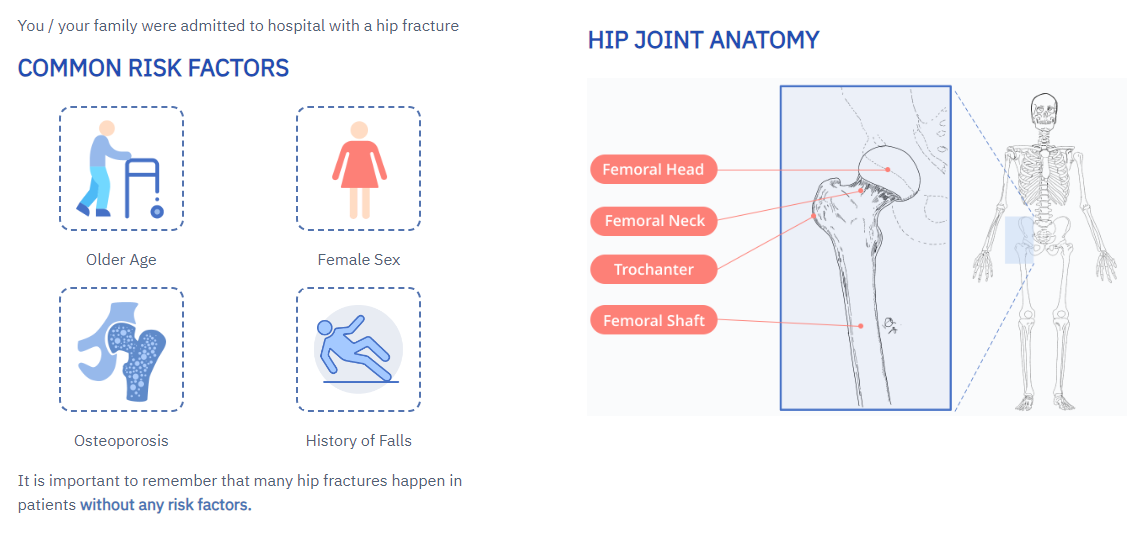


1. **Hip Fracture Treatment**


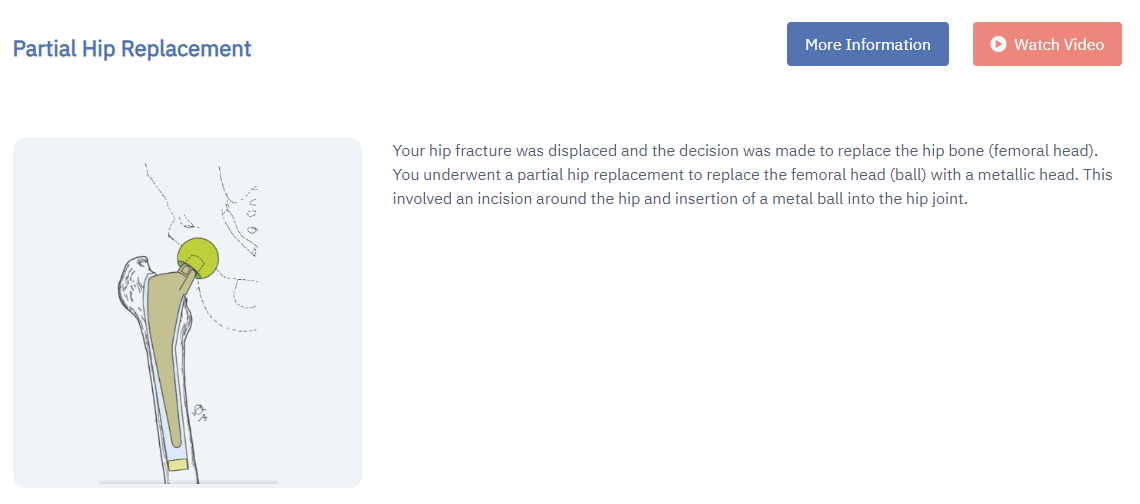


1. **Your Individual Risk: Risk of Serious Complications**
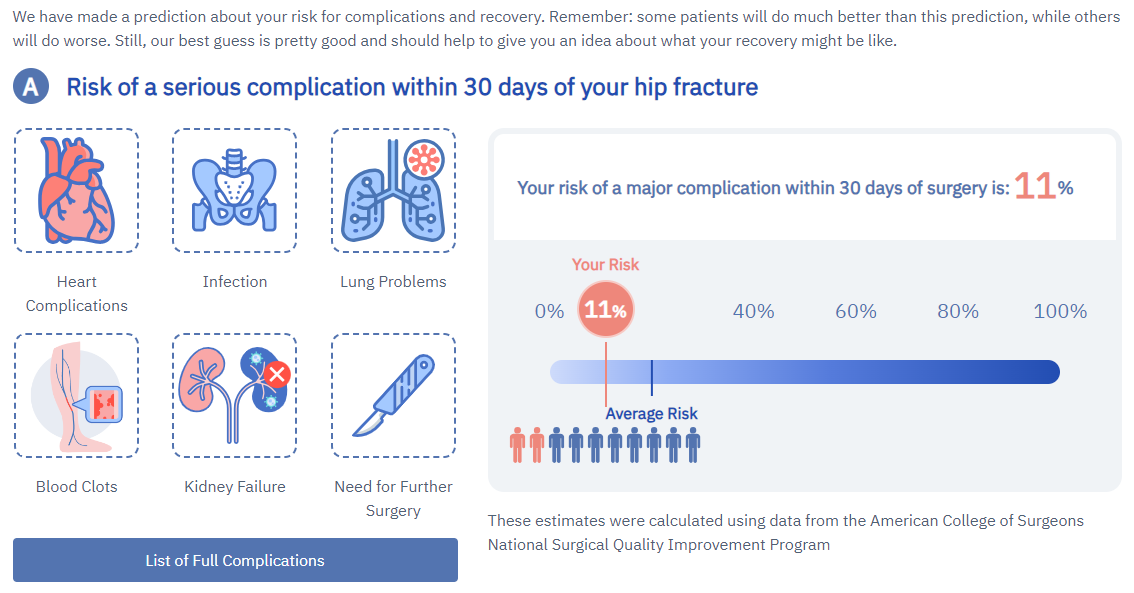

2. **Your Individual Risk: Risk of Serious Complications**


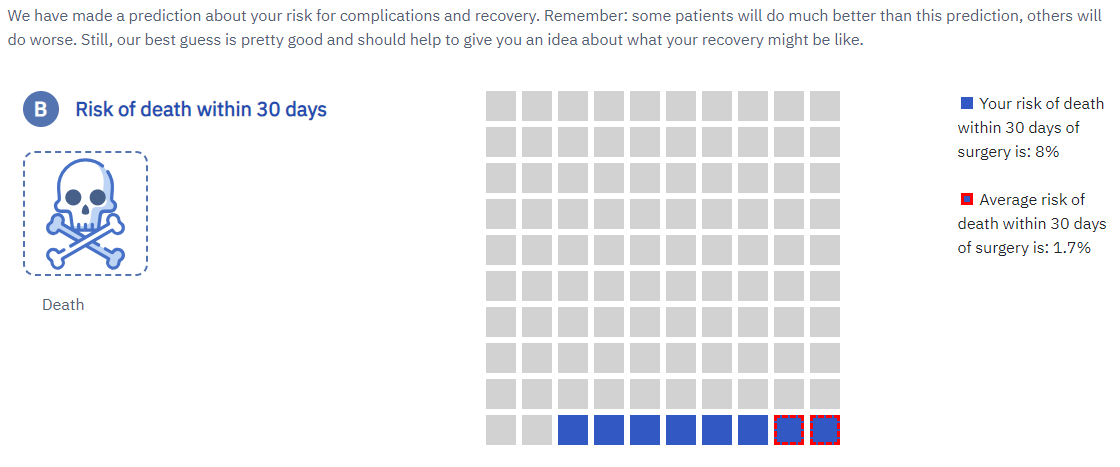


**5. What’s Next?**


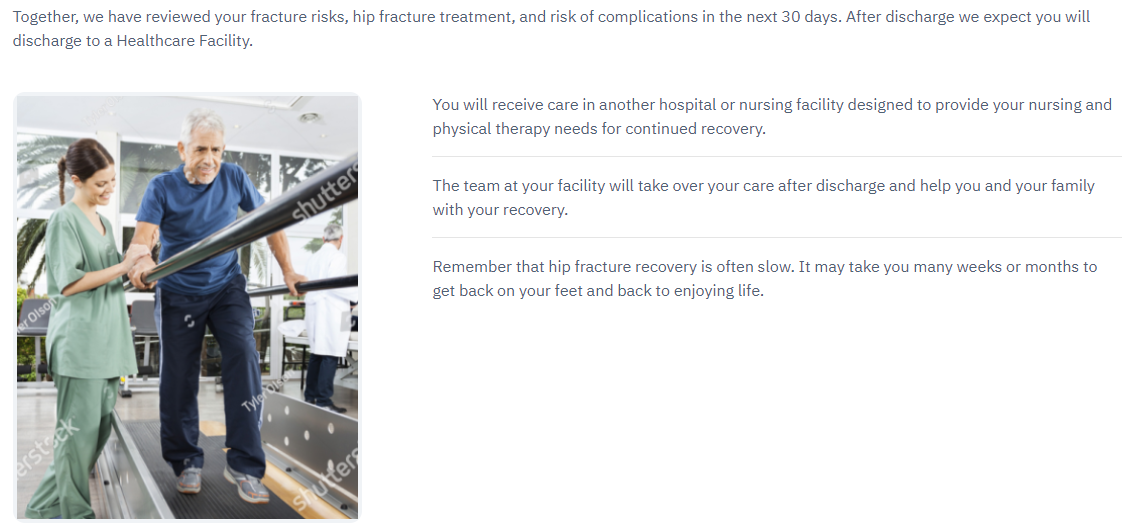


**Appendix 3: Simulated Patient Case**

**Name: Susie Smith**  **MRN:**  **000000S**  **DOB: 6/21/1928**

**CC:** mechanical fall with R hip pain

**HPI:** 93 yo female with PMHx of dementia who presents with her daughter after a mechanical fall at home. The cause of the fall is not clear but there was no evidence of loss of consciousness before or after the fall. She was up walking with her walker and seemed to just lose her balance. She then began having severe R hip pain, sharp and worse with movement.

**PMHx:**

Coronary artery disease  Mild Dementia

Atrial Fibrillation   CKD stage 3, baseline creatinine 1.4

Pulmonary hypertension Remote history of stroke

Essential Hypertension   Type 2 DM, diet controlled, A1C 6.1

Hyperlipidemia   Gout

Depression

**Past Surgical History:** hysterectomy, total knee arthroplasty, cholecystectomy, ventral hernia repair

**Home medications:**

Clopidogrel Allopurinol

Furosemide 20 daily Docusate

Escitalopram Lorazepam at bedtime

**Social History:**

Widowed, retired, lives with daughter in home, 3^rd^ grade education

Non-smoker, no alcohol use, no drug use

Uses walker, partially dependent in most ADLs and fully dependent in all IADLs

**PE:**

Temp 37.3 HR 76-93 BP 158-219/59-90 Resp 14-20  Ht 4’10” Wt 130 lb

Alert and oriented times 3

Lungs clear

Heart irregularly irregular, rate controlled, no murmur

Abdo soft, nontender

MSK no edema, R hip tender, externally rotated

**Labs:**

WBC 6.5 HGB 9.4 Plt 285

Na 145 K 5.0 Cl 111 CO2 22

BUN 37 (baseline 23) Cr 1.46 (baseline 1.4)

Hip Xray: R femoral neck fracture

Echo: EF 55-60%, septal motion suggests conduction abnormality, RVSP could not be measured, valvular thickening without stenosis

**Hospital Day #1:** Undergoes R hip hemiarthroplasty
